# Tai Chi and Yoga for Improving Balance on One Leg: A Neuroimaging and Biomechanics Study

**DOI:** 10.3389/fneur.2021.746599

**Published:** 2021-10-13

**Authors:** Xin-Peng Chen, Le-Jun Wang, Xiao-Qian Chang, Kuan Wang, Hui-Fang Wang, Ming Ni, Wen-Xin Niu, Ming Zhang

**Affiliations:** ^1^Shanghai YangZhi Rehabilitation Hospital (Shanghai Sunshine Rehabilitation Center), School of Medicine, Tongji University, Shanghai, China; ^2^Laboratory of Biomechanics and Rehabilitation Engineering, School of Medicine, Tongji University, Shanghai, China; ^3^Physical Education Department, Sport and Health Research Center, Tongji University, Shanghai, China; ^4^Department of Orthopaedics, Pudong New Area Peoples' Hospital, Shanghai University of Medicine and Health Sciences, Shanghai, China; ^5^Department of Biomedical Engineering, Faculty of Engineering, The Hong Kong Polytechnic University, Hong Kong, China

**Keywords:** yoga, one-leg stance, postural control, functional near-infrared spectroscopy, rambling and trembling, Taijiquan (T'ai Chi Ch'uan)

## Abstract

The one-leg stance is frequently used in balance training and rehabilitation programs for various balance disorders. There are some typical one-leg stance postures in Tai Chi (TC) and yoga, which are normally used for improving balance. However, the mechanism is poorly understood. Besides, the differences of one-leg stance postures between TC and yoga in training balance are still unknown. Therefore, the aim of the present study was to investigate cortical activation and rambling and trembling trajectories to elucidate the possible mechanism of improving one-leg stance balance, and compare the postural demands during one-leg stance postures between TC and yoga. Thirty-two healthy young individuals were recruited to perform two TC one-leg stance postures, i.e., right heel kick (RHK) and left lower body and stand on one leg (LSOL), two yoga postures, i.e., one-leg balance and Tree, and normal one-leg standing (OLS). Brain activation in the primary motor cortex, supplementary motor area (SMA), and dorsolateral prefrontal cortex (DLPFC) was measured using functional near-infrared spectroscopy. The center of pressure was simultaneously recorded using a force platform and decomposed into rambling and trembling components. One-way repeated-measures analysis of variance was used for the main effects. The relative concentration changes of oxygenated hemoglobin (ΔHbO) in SMA were significantly higher during RHK, LSOL, and Tree than that during OLS (*p* < 0.001). RHK (*p* < 0.001), LSOL (*p* = 0.003), and Tree (*p* = 0.006) all showed significantly larger root mean square rambling (RmRMS) than that during OLS in the medial–lateral direction. The right DLPFC activation was significantly greater during the RHK than that during the Tree (*p* = 0.023), OLB (*p* < 0.001), and OLS (*p* = 0.013) postures. In conclusion, the RHK, LSOL, and Tree could be used as training movements for people with impaired balance. Furthermore, the RHK in TC may provide more cognitive training in postural control than Tree and OLB in yoga. Knowledge from this study could be used and implemented in training one-leg stance balance.

## Introduction

Postural control during the one-leg stance is a common and important form of locomotion for people. It requires sensory systems, muscular activations, and passive dynamics (i.e., ligament and joint stiffness) to coordinate with the central nervous system (1). In addition, the narrow base of support makes it challenging to maintain balance in the one-leg stance (2). Thus, a one-leg stance is frequently used in balance training and rehabilitation programs for various balance disorders (3).

Tai Chi (TC) and yoga are both mind–body therapies that have been widely used for health promotion in ancient China and India, and have become increasingly popular (4). Recent studies showed that TC and yoga could improve balance on one leg (5, 6). However, the mechanism underlying this improvement is poorly understood. There are some typical one-leg stance postures in TC and yoga; the positions of the hands and feet are also quite different between postures, which can lead to different postural demands when performing (7, 8). Thus, the postural demands during one-leg stance postures between TC and yoga were also compared.

Previous studies have investigated the plantar pressure distribution during the one-leg stance of TC, and surface electromyography during the one-leg stance of yoga (9, 10). However, only one-leg stance postures in terms of the peripheral control system were investigated in previous studies. The efficiency of postural control processes during one-leg standing could also depend on complex cortical control. Herold et al. (11) found that the primary motor cortex (M1) and supplementary motor area (SMA) were activated during an unstable standing in healthy young adults. Luks et al. (12) highlighted the involvement of the dorsolateral prefrontal cortex (DLPFC), which could allocate attentional resources to maintain postural control. Among the various neuroimaging techniques, functional near-infrared spectroscopy (fNIRS) can detect differences in the absorption spectra of oxygenated hemoglobin vs. deoxygenated hemoglobin in the near-infrared spectrum range. Recent systematic reviews have highlighted the applicability of fNIRS as a neuroimaging technique to quantify the cortical control of static and dynamic forms of balance (13). However, there is still insufficient information regarding cortical activation during the one-leg stance postures of TC and yoga.

The center of pressure (COP) and its derived indicators are frequently used to evaluate postural control during one-leg standing (2). Zatsiorsky and Duarte (14) decomposed COP displacements into two components: rambling and trembling. Rambling represents the migration of the reference points in which the body is instantaneous in equilibrium and is under subcortical and cortical control, whereas trembling is associated with the action of spinal reflexes and the passive mechanical properties of the muscles (15). The cerebral cortex may regulate the excitability of the subcortical structure, spine, and muscle to maintain balance and postural stability according to physical demands (16). However, the correlation between cortical activation and sway parameters during one-leg stance postures is still unknown.

Therefore, the aim of the present study was to investigate cortical activation and rambling and trembling trajectories to elucidate the possible mechanism of improving one-leg stance balance, and to compare the postural demands during one-leg stance postures between TC and yoga. It was hypothesized that greater cortical activation and sway parameters might be related to improved one-leg stance balance, and that postural demands differ between the one-leg stance postures of TC and yoga.

## Materials and Methods

### Participants

Thirty-two healthy young individuals (12 men, 20 women, age: 22 ± 1 years, height: 1.63 ± 0.07 m, and weight: 54 ± 11 kg) were recruited in this study. All of them had dominant right legs, which were determined individually by asking which leg they would use to kick a ball as far as possible (17). The participants in the study had no TC or yoga experience, no disorders influencing balance capacity, and no history of injury with residual symptoms (e.g., pain, or “giving way” sensations) in the lower extremities within the last year.

This study was approved by the Ethics Committee of Shanghai University of Sports, and all subjects signed an approved informed consent form before participating.

### Experimental Design

**Figure 1:**
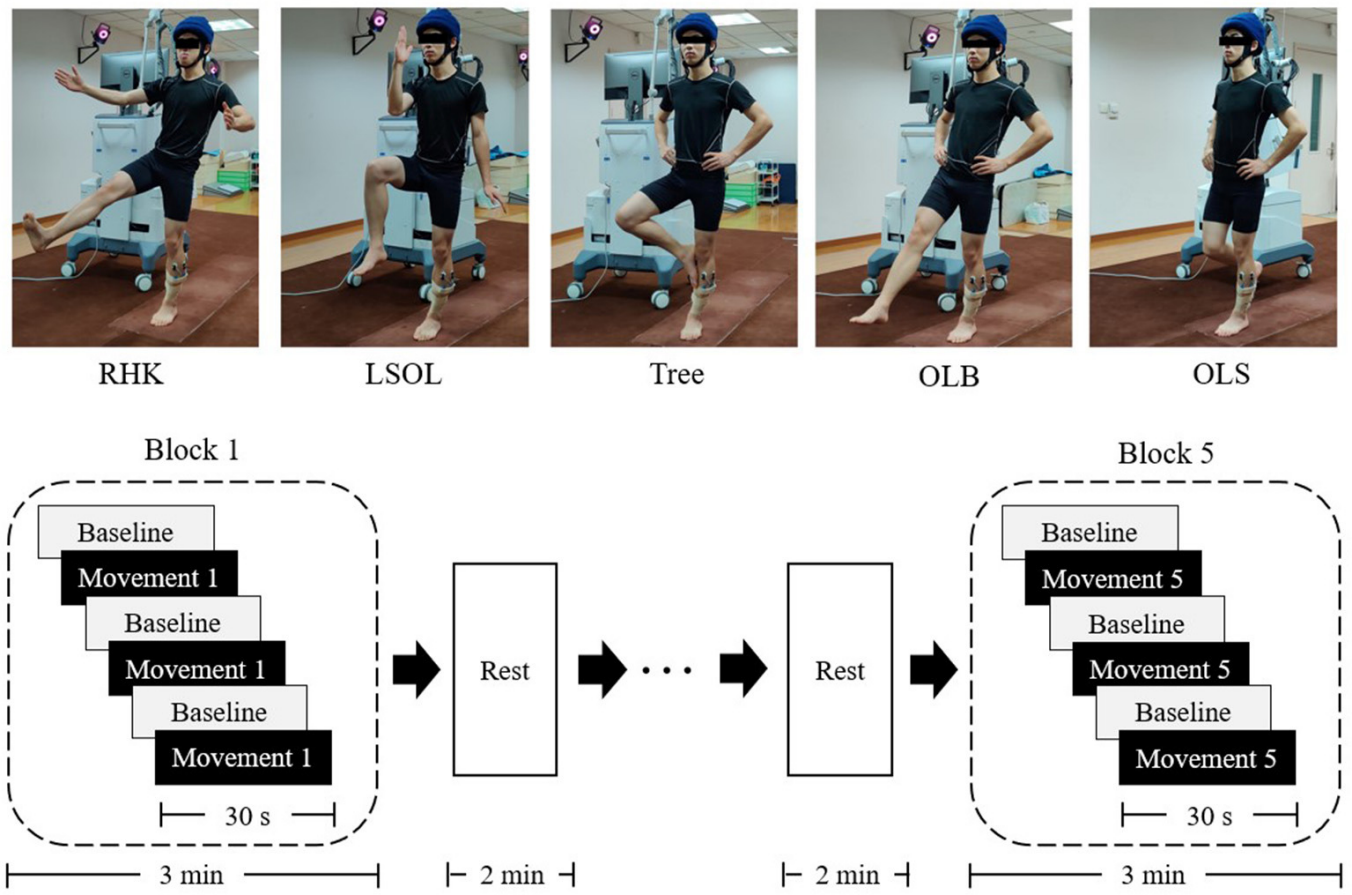
Five one-leg stance postures and experimental procedure. Five one-leg stance postures are shown in the upper panel. RHK, right heel kick; LSOL, left lower body and stand on one leg; OLB, one-leg balance; OLS, normal one-leg standing. Experimental procedure is shown in the lower panel. Baseline (gray rectangles): bipedal standing for 30 s; movement (black rectangles): preforming one-leg stance posture for 30 s.

There are many one-leg stance postures in TC and yoga. In this study, two TC one-leg stance postures, i.e., right heel kick (RHK) and left lower body and stand on one leg (LSOL), and two yoga postures, i.e., one-leg balance and Tree (Figure 1, upper panel) were measured, for most of the one-leg stance movements in TC (6, 18) and yoga (19) are based on these postures. In addition, brain activation and postural sway were measured in normal one-leg standing (OLS) with eyes open, which are regarded as the basic demands of balancing on one leg, for comparison with the selected postures in TC and yoga.

Prior to the experiment, the participants practiced the selected postures under the guidance of a coach (5 years of TC teaching experience and 3 years of yoga teaching experience) to ensure that the postures could be successfully performed three times.

This study had a block design, and each participant was required to perform five tasks randomly. There would be a 2-min rest between tasks. Each task condition was completed in a single block, which consisted of three repeated trials. Each trial lasted for 60 s, including 30 s of movement and 30 s of bipedal standing before tasks to serve as the baseline (Figure 1, lower panel). Participants were instructed to maintain a stable head position and to fixate on a visual target positioned in front of them above the eye level. The measurements were performed in a quiet room. All participants completed the five postures, with a success rate of 93.8%. Two participants lost their balance during the tasks, and they were retested.

### Functional Near-Infrared Spectroscopy Data Acquisition and Processing

**Figure 2:**
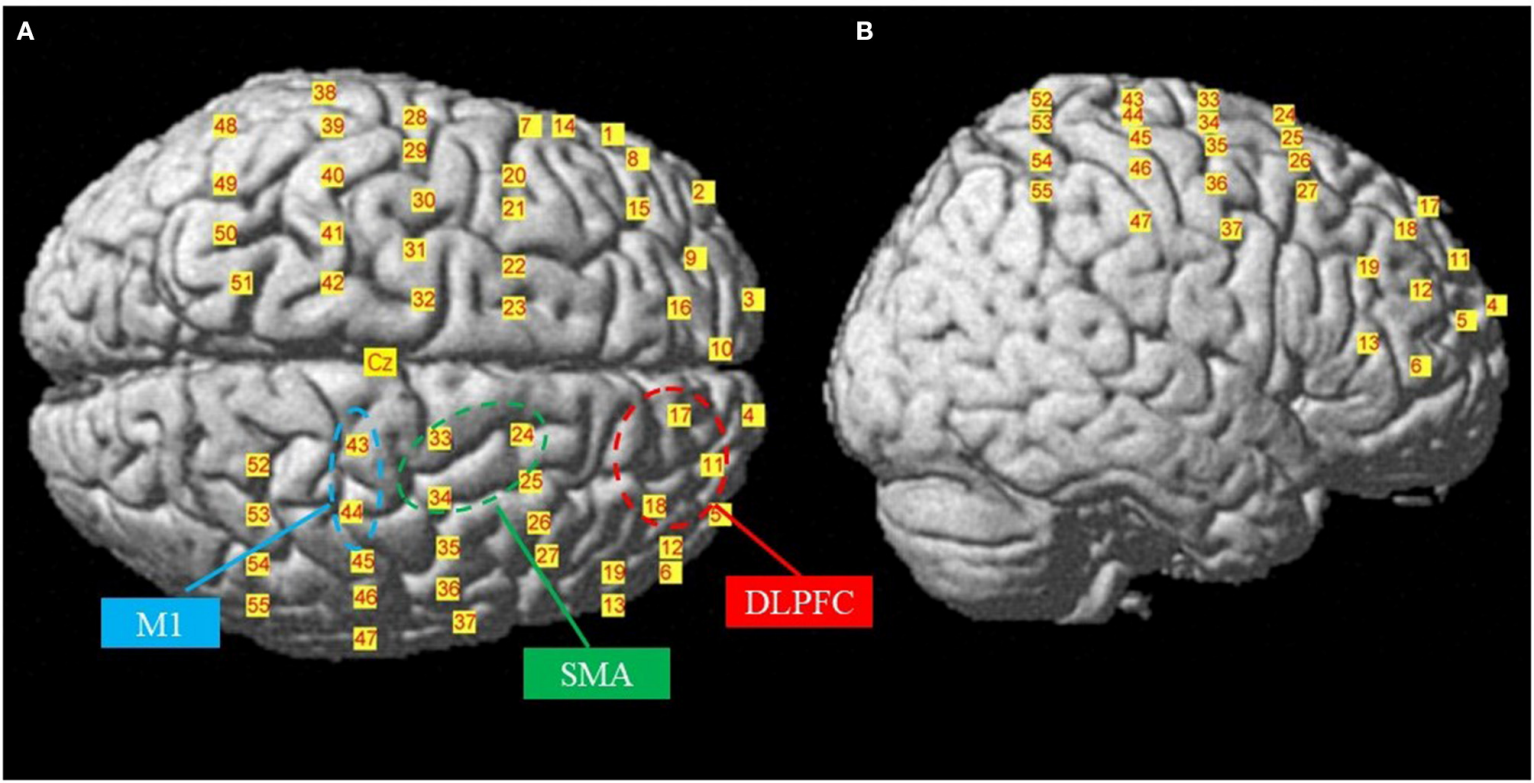
The locations of the channels. Top **(A)** and right-lateral **(B)** views of the locations of the channels in the MNI brain. SMA, supplementary motor area; M1, primary motor cortex; DLPFC, dorsolateral prefrontal cortex.

Multichannel tissue oxygenation monitoring technology with continuous waves (NirSmart, Danyang Huichuang Medical Equipment Co., Ltd., Jiangsu, China) was used in this study. The overall optical system consisted of 17 emitters (740, 808, and 850 nm) and 19 detectors with a sampling frequency of 10 Hz, arranged on the heads of the participants, with 3-cm inter-optode spacing. The Cz position was located at the intersection of CH32, CH33, CH42, and CH43, and the Fz position was located at the middle point of CH16 and CH17 (Figure 2). The probe locations were measured using a 3D position-measuring system (FASTRAC; Polhemus, Colchester, VT, USA) (Supplementary Table 1). The acquired coordinates were transformed into MNI coordinates and further projected to the MNI standard brain template using the NIRS_SPM toolbox (20). Based on these data, a reference brain database was built (21), and the brain locations corresponding to each channel were identified. This configuration allowed the quantification of activity changes in the right DLPFC (CH 11, 17, and 18), right SMA (CH 24, 32, and 33), and right M1 (CH 43 and 44). In this study, only the right hemisphere was analyzed because all one-leg stance postures involved were supported by the left leg.

The fNIRS data were preprocessed using MATLAB 2014a (The MathWorks Inc., United States) and the MATLAB-based fNIRS-processing package HomER2 (22). The raw intensity data were converted to optical density changes, and then the spline interpolation algorithm was used to correct motion artifacts caused by head movements during data acquisition (23). Then, a bandpass filter between 0.01 and 0.2 Hz was used to remove the effects of physiological noise and drift. Finally, the optical density was converted into relative concentration changes of oxygenated hemoglobin (ΔHbO) and deoxygenated hemoglobin based on the modified Beer–Lambert law (24). In this study, ΔHbO signals were adopted as an indicator of hemodynamic response because they are more sensitive than deoxyhemoglobin to regional cerebral blood flow (25). For each trial, the average concentration of oxygenated hemoglobin was calculated for 15 s before the task (referred to as the baseline) and 5–25 s during the task. Each baseline concentration was subtracted from the average concentration during task performance to evaluate ΔHbO (26). Then the results of the three trials were averaged for each region of interest (ROI).

### Kinetics Data Acquisition and Processing

An AMTI force platform (Advanced Medical Technology, Inc., Watertown, MA, USA) was used to record the ground reaction force and COP in both the anterior–posterior (AP) and medial–lateral (ML) directions during the one-leg stance postures. The force platform collected signals with a sampling frequency of 1,000 Hz and synchronized it with the fNIRS system.

**Figure 3:**
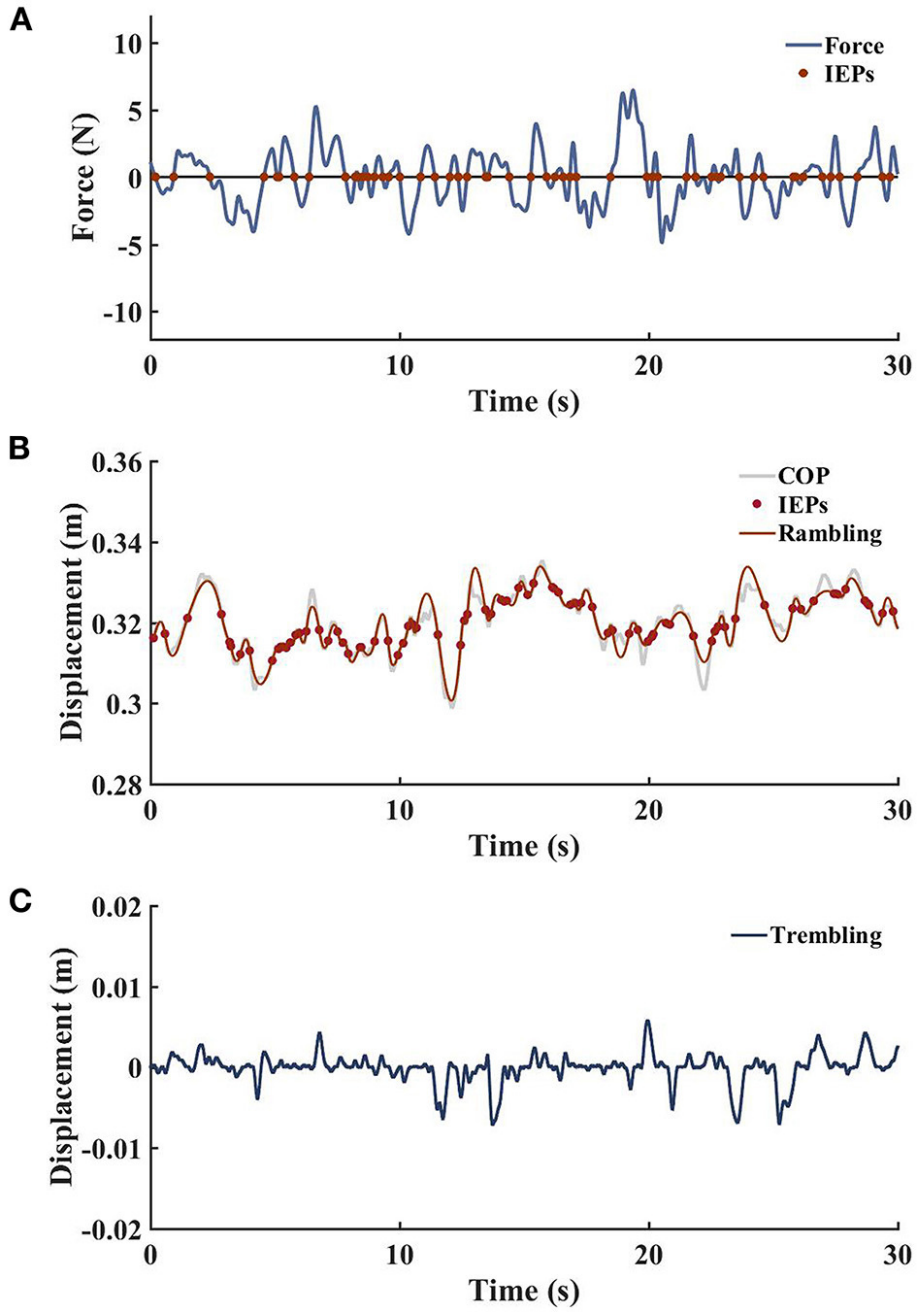
Sequence of operations used for center of pressure (COP) trajectory decomposition. **(A)** Instant equilibrium points (IEPs) occur when the horizontal force component is zero (or very close to zero). **(B)** In the COP displacement data, the COP positions at the instants (IEPs) are located, and the spline interpolated rambling trajectory is shown. **(C)** Trembling trajectory, the difference between COP and interpolated rambling trajectories.

The ground reaction force and COP data were processed using MATLAB 2014a (The MathWorks Inc., USA). The raw data were filtered using a fourth-order Butterworth low-pass filter with a cutoff of 10 Hz. After filtering and detrending the COP data, the rambling and trembling decomposed from the COP trajectories in the ML and AP directions were calculated according to the statistical method described by Zatsiorsky and Duarte (14, 15). An instant equilibrium point (IEP) occurs when the horizontal force component is zero or very close to zero, implying that the ground reaction force is vertical at that instant (Figure 3A). A cubic spline was used to reconstruct an IEP trajectory from these points, which was the rambling trajectory (Figure 3B). The trembling was estimated as the deviation of the COP trajectory from the IEPs (Figure 3C). Then, the root mean squares of the rambling (Rm_RMS_) and trembling (Tm_RMS_) trajectories and the mean power frequencies of the rambling (Rm_F_) and trembling (Tm_F_) trajectories were calculated. All indicators were calculated separately in the ML and AP directions.

### Statistical Analysis

A statistical analysis was conducted using SPSS 22 (IBM SPSS Statistics, Chicago, IL, USA). The normality of distribution (Shapiro–Wilk test) and homoscedasticity of data (Levene's test) were verified before the application of the parametric tests in the case of a normal distribution. One-way repeated-measures analysis of variance (ANOVA) was used for the comparison of the five tasks, while the Friedman test was conducted in the absence of normality. The Bonferroni *post-hoc* test was used for multiple comparisons. To examine the relationship between cortical activation and postural sway, Pearson's correlation coefficients were calculated between ROIs and the rambling and trembling parameters. The level of statistical significance was set at *p* < 0.05.

## Results

### Functional Near-Infrared Spectroscopy Data

**Figure 4:**
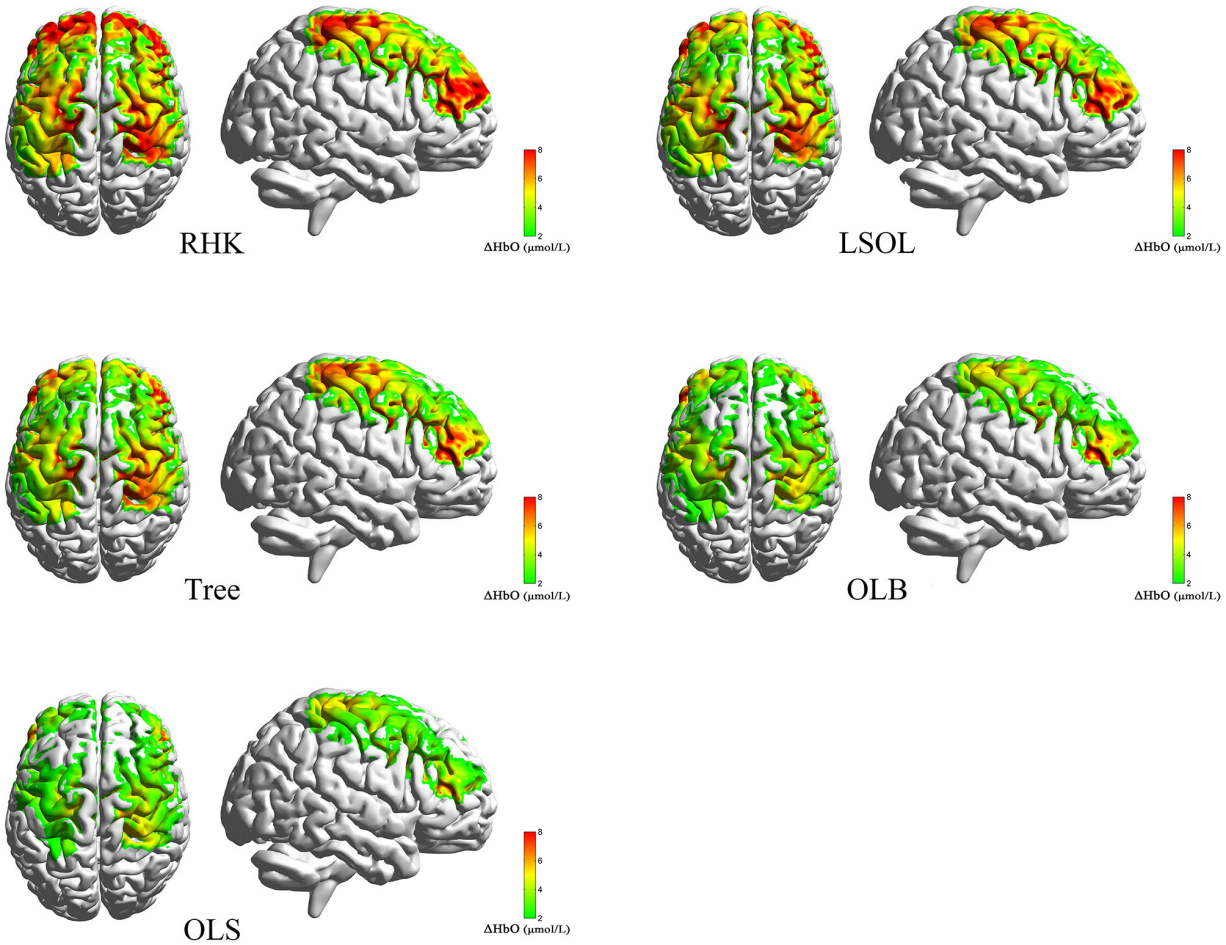
Averaged concentration changes of oxyhemoglobin (ΔHbO) during postures. RHK, right heel kick; LSOL, left lower body and stand on one leg; OLB, one-leg balance; OLS, normal one-leg standing.

**Figure 5:**
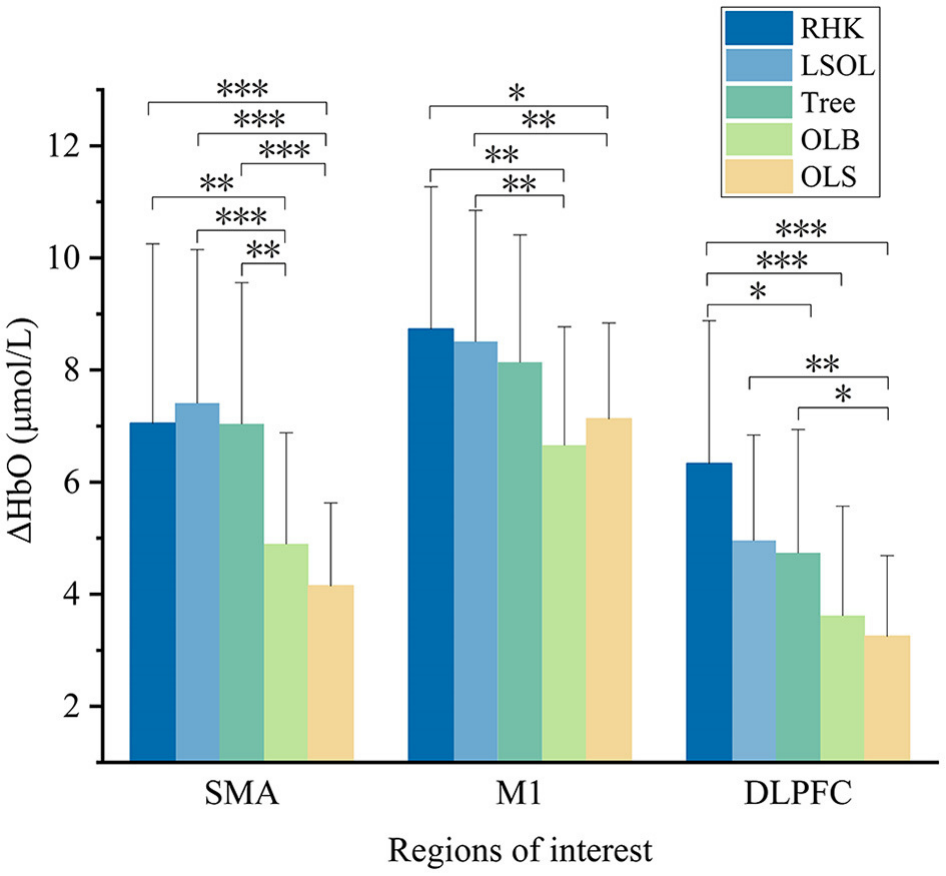
The significant differences of cortical activation between postures. RHK, right heel kick; LSOL, left lower body and stand on one leg; OLB, one-leg balance; OLS, normal one-leg standing; SMA, supplementary motor area; M1, primary motor cortex; DLPFC, dorsolateral prefrontal cortex; Significant differences between postures: *p < 0.05, **p < 0.01, and ***p < 0.001.

The mean ΔHbO values of the five tasks (RHK, LSOL, Tree, OLB, and OLS) are shown in Figure 4. The results of one-way repeated-measures ANOVA showed significant main effects on all ROIs in the right SMA [*F*_(4, 28)_ = 16.749; *p* < 0.001; η^2^ = 0.351], right M1 [*F*_(4, 28)_ = 6.806; *p* < 0.001; η^2^ = 0.180], and right DLPFC [*F*_(4, 28)_ = 13.811; *p* < 0.001; η^2^ = 0.308]. The results with significant differences in the *post-hoc* analysis are shown in Figure 5. The SMA had significantly greater activation during the RHK, LSOL, and Tree postures than OLS (*p* < 0.001). The ΔHbO in M1 was significantly greater during RHK than during OLB (*p* = 0.007) and OLS (*p* = 0.049), and the ΔHbO in M1 was significantly greater during LSOL than during OLB (*p* = 0.004) and OLS (*p* = 0.005). The right DLPFC activation was significantly greater during the RHK than during the Tree (*p* = 0.023), OLB (*p* < 0.001), and OLS (*p* = 0.013) postures.

### Kinetics Data

There were significant effects of different tasks on both the Rm_RMS_ and Rm_F_ in the AP direction, Rm_RMS_: [*F*_(4, 28)_ = 2.879; *p* = 0.025; η^2^ = 0.085]; Rm_F_: [*F*_(4, 28)_ = 10.507; *p* < 0.001; η^2^ = 0.253], and ML direction, Rm_RMS_: [*F*_(4, 28)_ = 8.386; *p* < 0.001; η^2^ = 0.213]; Rm_F_: [*F*_(4, 28)_ = 3.076; *p* = 0.019; η^2^ = 0.090]. The results with significant differences in the *post-hoc* analysis are shown in Table 1. RHK showed a significantly higher Rm_F_ than LSOL (*p* = 0.002), Tree (*p* = 0.015), and OLB (*p* = 0.003) in the AP direction. RHK (*p* < 0.001), LSOL (*p* = 0.003), and Tree (*p* = 0.006) showed significantly greater Rm_RMS_ than OLS in the ML direction. For trembling, the results showed significant main effects on Tm_RMS_ in the ML direction [*F*_(4, 28)_ = 5.675; *p* < 0.001; η^2^ = 0.155]. *Post-hoc* pairwise comparisons revealed that the Tm_RMS_ of RHK was significantly larger than that of OLS in the ML direction (*p* = 0.002).

**Table 1:** Rambling and trembling parameters during one-leg stance postures of Tai Chi and yoga.

	**RHK**	**LSOL**	**Tree**	**OLB**	**OLS**
Anterior–posterior direction
Rm_RMS_ (mm)	7.91 ± 1.21	7.73 ± 0.98	8.00 ± 1.24^d^	7.30 ± 1.22	7.81 ± 1.21
Tm_RMS_ (mm)	1.98 ± 0.27	1.81 ± 0.25	1.87 ± 0.31	1.82 ± 0.38	1.85 ± 0.31
Rm_F_ (Hz)	0.50 ± 0.10^b^^c^^d^^e^	0.45 ± 0.11	0.46 ± 0.09^e^	0.45 ± 0.10	0.42 ± 0.10
Tm_F_ (Hz)	1.21 ± 0.21	1.21 ± 0.28	1.15 ± 0.18	1.14 ± 0.24	1.12 ± 0.22
Medial–lateral direction
Rm_RMS_ (mm)	7.44 ± 0.77^d^^e^	7.16 ± 0.87^e^	7.30 ± 1.17^e^	6.90 ± 0.84	6.61 ± 0.75
Tm_RMS_ (mm)	2.32 ± 0.33	2.15 ± 0.35	2.18 ± 0.38	2.14 ± 0.38	1.99 ± 0.36^a^
Rm_F_ (Hz)	0.64 ± 0.12	0.61 ± 0.08	0.59 ± 0.10	0.58 ± 0.12	0.60 ± 0.13
Tm_F_ (Hz)	1.18 ± 0.31	1.10 ± 0.21	1.07 ± 0.21	1.05 ± 0.20	1.13 ± 0.27

*RHK, right heel kick; LSOL, left lower body and stand on one leg; OLB, one-leg balance; OLS, normal one-leg standing; Rm_RMS_, root mean square of rambling; Tm_RMS_, root mean square of trembling; Rm_F_, mean power frequency of rambling; Tm_F_, mean power frequency of trembling*.

a *Statistically significant difference from RHK (p < 0.05)*.

b *Statistically significant difference from LSOL (p < 0.05)*.

c *Statistically significant difference from Tree (p < 0.05)*.

d *Statistically significant difference from OLB (p < 0.05)*.

e *Statistically significant difference from OLS (p < 0.05)*.

### Correlation Analysis

**Figure 6:**
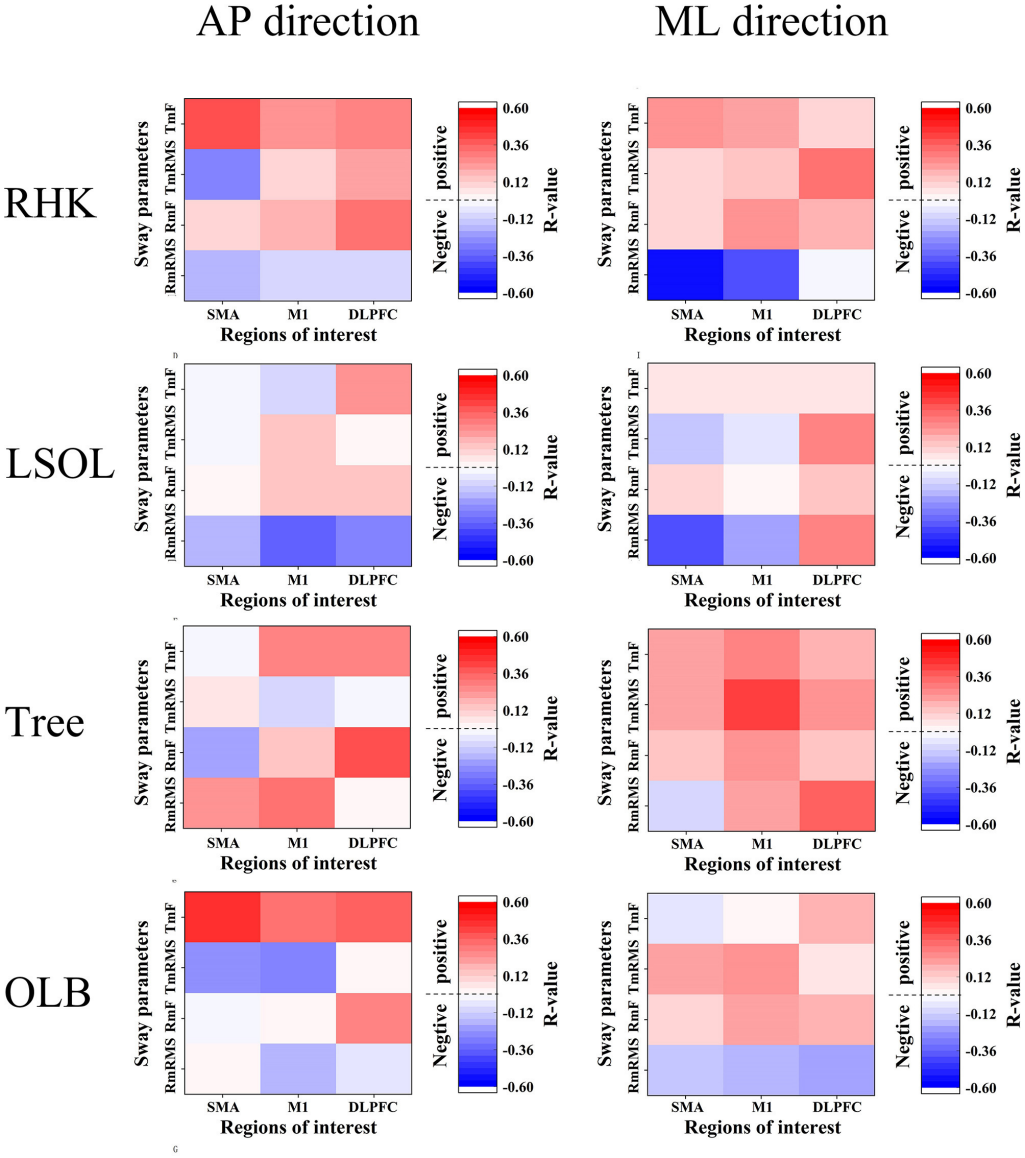
Correlations between sway parameters and brain activation. RHK, right heel kick; LSOL, left lower body and stand on one leg; OLB, one-leg balance; SMA, supplementary motor area; M1, primary motor cortex; DLPFC, dorsolateral prefrontal cortex; Rm_RMS_, root mean square of rambling; Tm_RMS_, root mean square of trembling; Rm_F_, mean power frequency of rambling; Tm_F_, mean power frequency of trembling.

**Figure 7:**
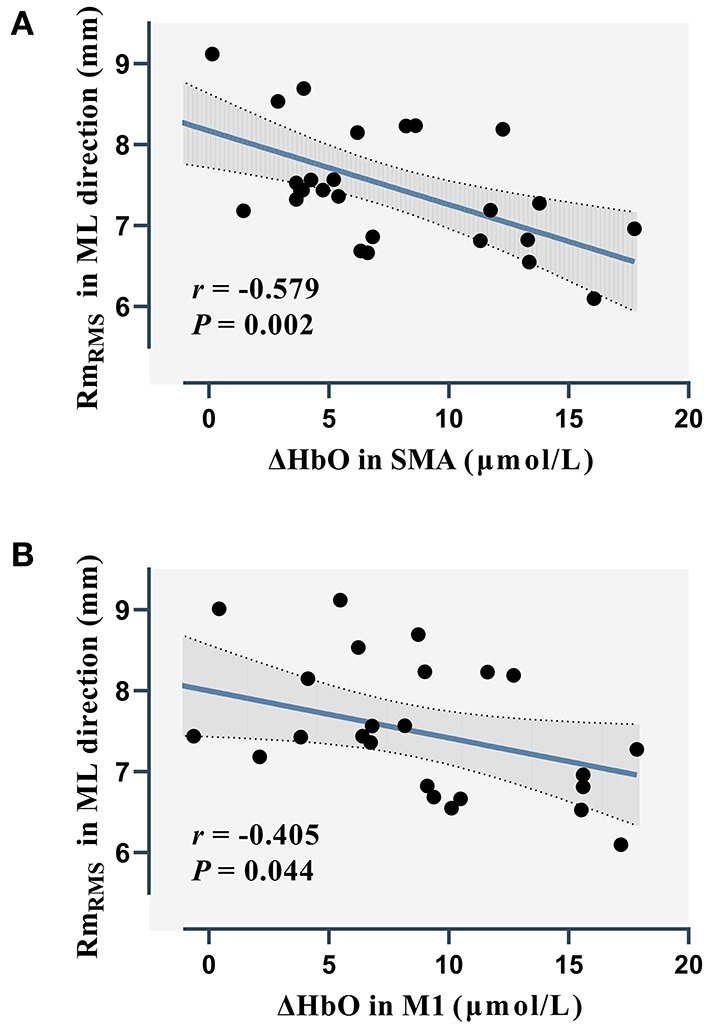
Correlations between rambling parameters and brain activation in the SMA **(A)** and M1 **(B)** during RHK. ΔHbO, relative concentration changes of oxygenated hemoglobin; Rm_RMS_, root mean square of rambling; ML, medial-lateral; SMA, supplementary motor area; M1, primary motor cortex.

Figure 6 shows the results of the correlation between cortical activity and rambling and trembling parameters. Significant negative correlations were noted between ΔHbO values in the SMA (*r* = −0.579; *p* = 0.002) and M1 (*r* = −0.405; *p* = 0.044) and Rm_RMS_ in the ML direction during RHK (Figures 7A,B). In the LSOL posture, a negative correlation was found between the SMA and Rm_RMS_ in the ML direction (*r* = −0.436; *p* = 0.022).

## Discussion

In this study, cortical activation, rambling, and trembling parameters were observed during five one-leg stance postures. Postural demands during four postures in TC and yoga were compared with those during OLS. The cortical activation and sway parameters during OLS are regarded as the basic demands of balancing on one leg.

The SMA had significantly greater activation during the RHK, LSOL, and Tree postures than that during OLS. Solis-Escalante et al. (16) used EEG-based analysis of cortical oscillatory dynamics during the preparation and execution of balance responses, and found that higher postural demand was reflected in stronger power modulations during preparatory balance control. In addition, the SMA is also involved in preparation for foot movement (27). During the one-leg stance, the ankle strategy appears to be dominant in the control of unperturbed standing. In the ankle strategy, balance is maintained or restored mainly by movement of the body around the ankle as a single inverted pendulum to adapt to body sway (28). Thus, the greater activation of the SMA may be related to improved one-leg stance balance. In addition, RHK, LSOL, and Tree showed significantly larger Rm_RMS_ values than OLS in the ML direction. According to the hypothesis of Zatsiorsky and Duarte (14, 15), the rambling component is proposed to contribute to continuous postural adjustments made by the supraspinal central nervous system while seeking for equilibrium to adapt to body sway. A larger sway of rambling trajectories suggests that the supraspinal central nervous system was responsible for maintaining one-leg stance balance on a larger scale in the ML direction. Nakamura et al. (29) found that an increase in the displacement of body sway not only increased the magnitude of the electromyography, but also increased the number of muscles used in the lower extremities. Therefore, combining the SMA activation and Rm_RMS_ parameters, it suggested that RHK, LSOL, and Tree may be related to improving one-leg stance balance in the ML direction. However, there were no significant differences between OLB and OLS. This result suggests that the two postures can be replaced by each other for one-leg stance balance training.

The M1 is an important structure in motor control. It receives and processes inputs from almost all cortical areas implicated in motor control, including the premotor cortex, SMA, and somatosensory cortex, and it sends motor commands through the corticospinal tract to modulate postural control (30). The results also showed that ΔHbO in M1 during RHK and LSOL increased significantly compared with that during OLS. A previous study found that M1 was not only involved in static and dynamic balance control (31, 32), but it also adapted in response to balance training. Another study found that rapid and specific gray matter thickness increased in M1 after 1 h of practice in a complex balancing task (33). Thus, the greater activation of M1 may be related to improved one-leg stance balance. However, the activation of M1 during the Tree showed no significant difference from that during OLS, which was not in line with the results of SMA activation. This result suggested that not all cortical area activation was greatly associated with more demanding postural tasks. Previous studies have found that the SMA is part of the indirect locomotor pathway, which is activated during complex motor tasks or complex general tasks (34, 35). In contrast, the M1 is considered to be part of the direct locomotor pathway, which is activated during more automatic tasks (36). During the RHK and LSOL postures, the upper limbs and right foot were required to be suspended in the air and remained still. It was quite challenging for the central nervous system to coordinate the movement of the upper and lower limbs while maintaining one-leg stance balance.

In terms of trembling, the results showed a significantly larger Tm_RMS_ during RHK than during OLS in the ML direction. The trembling component reflects the peripheral mechanisms of the postural control system, such as the passive mechanical properties of the muscles, ligaments, and joints (37). In addition, a previous study revealed the existence of a large negative correlation between horizontal force and trembling (14). Hence, each deviation of the COP from the interpolated IEP trajectory is associated with a horizontal force that tends to reduce the deviation. The horizontal force directly stimulates peripheral sensory receptors in muscles and ligaments, and the generated sensory information is sent to the central nervous system, which is used for postural adjustment (1). Compared with OLS, the RHK may be a more efficient training movement for people with postural stability deficits after anterior cruciate ligament injury (38) or ankle sprain (39).

In this study, the postural demands of four one-leg stance postures in TC and yoga were also compared. The results showed that the activation in the DLPFC during RHK was significantly greater than that during LSOL, Tree, and OLB. The DLPFC is involved in allocating attentional resources to maintain postural control and integrating external information with information on body position (12). It suggested that greater attentional resources were required for postural stabilization during RHK. A previous study found that transcranial direct current stimulation over the DLPFC reduced the dual task cost, and improved balance performance in cognitive–motor dual-task situations involving a serial subtraction task in young adults (40). Thus, the RHK may provide more cognitive training in postural control than Tree and OLB in yoga. This finding was in line with a previous study. Xie et al. (41) explored the effects of TC on the effective connection of the prefrontal, motor, and occipital cortices and found that TC training enhanced brain functional connections, thus indicating the ability of TC to improve cognition.

The process of supraspinal control involves the cerebral cortex and subcortical structures, such as the cerebellum, the basal ganglia, and the brainstem (42). In the current study, RHK showed a significantly higher Rm_F_ than other postures in the AP direction. In a previous study, postural sway was observed in challenging motor and sensory conditions, and it was found that the mean power frequencies of rambling increased in the AP direction (43). The increasing mean power frequencies of rambling might be caused by supraspinal control pathways modulated during challenging conditions (44). Furthermore, significant negative correlations were explored between SMA and M1 activation and Rm_RMS_ in the RHK in the ML direction. Taube et al. (31) investigated changes in corticospinal and spinal excitability after balance training and found a negative correlation between cortical excitability and stance stability, suggesting that supraspinal adaptations mainly contributed to improved balance performance following training. Thus, our results may suggest that the subcortical structures are mainly responsible for postural control to meet higher postural demands during RHK, and they are involved in the automatic process of postural control, including balance adjustment and muscle tone regulation (35). However, significant negative correlations were not observed in the Tree and OLB postures. The absence of correlations may be related to different supraspinal control mechanisms.

## Limitations

The limitations of this study must be acknowledged. In this study, cortical activation and postural sway in one-leg stance postures were investigated. However, visual input is an important part of sensory integration during the one-leg stance postures. In further studies, visual contributions in postural control need to be clarified. Second, all the participants recruited were young healthy subjects. Further studies need to take the elderly or people with impaired balance due to neurological and musculoskeletal disorders into consideration. Moreover, the beneficial effects need to be identified by long-term intervention based on the one-leg stance postures.

## Conclusion

In conclusion, the RHK, LSOL, and Tree postures exhibited greater SMA activation and postural sway than that during OLS. These findings may be related to the mechanism of TC and yoga in improving one-leg stance balance. The RHK, LSOL, and Tree could be used as training movements for people with impaired balance. Furthermore, the RHK may provide more cognitive training in postural control than Tree and OLB in yoga. This study provided a more in-depth understanding of postural control in different traditional exercise training in one-leg stance using fNIRS and COP indicators in the force platform, and knowledge from this study could be used and implemented in training one-leg stance balance.

## Data Availability Statement

The raw data supporting the conclusions of this article will be made available by the authors, without undue reservation.

## Ethics Statement

The studies involving human participants were reviewed and approved by Ethics Committee of Shanghai University of Sports. The patients/participants provided their written informed consent to participate in this study. Written informed consent was obtained from the individual(s) for the publication of any potentially identifiable images or data included in this article.

## Author Contributions

L-JW, X-QC, H-FW, KW, W-XN, and MZ conceptualized this study. X-PC, L-JW, X-QC, and MN designed the methodology for the study. X-PC and X-QC participated in the data acquisition and data curation. X-PC, L-JW, X-QC, H-FW, and MN analyzed and interpreted the data. X-PC drafted the manuscript. KW, W-XN, and MZ critically revised the manuscript for content. All authors contributed to the article and approved the submitted version.

## Funding

This work was supported by the National Natural Science Foundation of China (11732015/32071308/31900942) and the Natural Science Foundation of Shanghai (20ZR1452600).

## Conflict of Interest

The authors declare that the research was conducted in the absence of any commercial or financial relationships that could be construed as a potential conflict of interest.

## Publisher's Note

All claims expressed in this article are solely those of the authors and do not necessarily represent those of their affiliated organizations, or those of the publisher, the editors and the reviewers. Any product that may be evaluated in this article, or claim that may be made by its manufacturer, is not guaranteed or endorsed by the publisher.
